# Associations between Systemic and Dental Diseases in Elderly Korean Population

**DOI:** 10.3390/medicina60101693

**Published:** 2024-10-15

**Authors:** Se Hoon Kahm, SungEun Yang

**Affiliations:** 1 Department of Dentistry, Eunpyeong St. Mary’s Hospital, College of Medicine, The Catholic University of Korea, Seoul 03312, Republic of Korea; implant@catholic.ac.kr; 2 Department of Conservative Dentistry, Seoul St. Mary’s Dental Hospital, College of Medicine, The Catholic University of Korea, Seoul 06591, Republic of Korea

**Keywords:** systemic disease, dental disease, multimorbidity, electronic medical record, clinical data warehouse

## Abstract

*Background and Objectives:* Modernization and population aging have increased the prevalence of systemic diseases, such as diabetes and hypertension, which are often accompanied by various dental diseases. Our aim was to investigate associations between common dental conditions and major systemic diseases in an elderly Korean population. *Materials and Methods*: Utilizing electronic medical record data from 43,525 elderly patients, we examined the prevalence of systemic diseases (diabetes, hypertension, rheumatoid arthritis, osteoporosis, dementia) and dental conditions (caries, periodontal disease, pulp necrosis, tooth loss). The analysis focused on the correlations between these diseases. *Results*: Significant associations were found between systemic diseases and an increased prevalence of dental conditions. Patients with systemic diseases, especially those with multiple conditions, had higher incidences of periodontal disease and tooth loss. The correlation was particularly strong in patients with diabetes and rheumatoid arthritis. Interestingly, temporomandibular joint disorder was less frequent in this cohort. *Conclusions*: The findings highlight the importance of integrated dental care in managing systemic diseases in elderly populations. Enhanced dental monitoring and proactive treatment are essential due to the strong association between systemic diseases and dental conditions. Collaboration between dental and medical professionals is crucial for comprehensive care that improves health outcomes and quality of life for elderly patients.

## 1. Introduction

With rising life expectancies, the prevalence of systemic and oral diseases has increased, often interlinked in a detrimental cycle for those affected. The interplay between systemic conditions, like diabetes and cardiovascular diseases, and oral health issues, such as periodontitis, has been substantiated in prior research [1]. The association between dental caries and systemic diseases is well established, particularly with conditions like diabetes, obesity, and cardiovascular diseases [2]. Additionally, mental health disorders, including depression, have shown a connection to oral health [3].

Multimorbidity, commonly observed in patients with systemic diseases, tends to escalate with age, complicating treatment due to potential interactions between multiple prescribed medications [4]. These patients often experience shorter life expectancy, higher hospitalization rates, and a significantly reduced quality of life. The overlapping effects of various medications can exacerbate existing conditions, making treatment more difficult. These complexities can also lead to increased healthcare use, potential treatment failures, and diminished quality of life and productivity [5,6]. Notably, multimorbidity may intensify these issues more than single systemic diseases.

Dental health, intricately linked to overall wellbeing, can deteriorate significantly in the presence of systemic diseases such as uncontrolled diabetes [7], increasing the risk for conditions like periodontal disease and tooth loss [8,9]. Individuals with four or more systemic diseases were found to have a 1.37 times higher risk of experiencing discomfort during mastication compared to healthy individuals, highlighting the significant impact of multimorbidity on oral function [10]. Recent studies have suggested a potential link between periodontitis and certain cancers, indicating that oral health issues may have broader systemic implications, further underscoring the importance of maintaining good oral hygiene and seeking timely dental care to mitigate these risks [11]. Previous studies have shown that tooth pain related to systemic diseases can lead to a significant decline in quality of life, leading to the emergence of the concept of “oral health-related quality of life” to describe this phenomenon. It has been widely suggested that maintaining good oral health is crucial not only for physical wellbeing but also for supporting a healthy aging process and preserving mental health [12,13]. Oral health, therefore, plays an integral role in promoting both overall health and psychological wellbeing as individuals age.

While previous research has explored the connections between individual systemic and dental diseases, the cumulative impact of multiple systemic conditions remains less studied. This study addresses that gap by examining prevalent systemic diseases—hypertension (HBP), diabetes mellitus (DM), rheumatoid arthritis (RA), osteoporosis (OPR), and dementia (DMT)—and their collective influence on dental health. Utilizing a retrospective database from two hospitals’ clinical data warehouses, we aim to extend current knowledge on the complex interrelations of dental and systemic health, contributing new perspectives to this intricate web of associations.

## 2. Materials and Methods

### 2.1. Patient Survey Overview and Study Subjects

Data for patients who visited the dental clinics of Seoul St. Mary’s Hospital and Eunpyeong St. Mary’s Hospital between 1 July 2011 and 30 June 2020 were extracted from the Catholic Medical Center Clinical Data Warehouse (CDW, Catholic University of Korea, Seoul, Republic of Korea). All data used were encoded and anonymized through the extraction system of CDW from the electronic medical record (EMR) data. Five systemic diseases and six dental diseases, which were frequently observed in the extracted data, were analyzed (Table 1). This study included patients diagnosed with systemic and dental diseases who had visited the hospital with the same diagnosis at least twice and received both diagnosis and treatment. Cases involving orthodontic treatment, orthognathic surgery, and tumors were excluded from the analysis of dental diseases. All diagnoses were identified, classified, and extracted using the International Classification of Diseases, 10th Revision (ICD-10) diagnostic codes to ensure standardization and accuracy in the data collection process.

This retrospective cohort study used EMR data retained by two hospitals. All data were extracted from the Clinical Data Warehouse (CDW), a centralized repository integrating clinical information from affiliated hospitals within the Catholic Medical Center network in Korea. The CDW serves as a comprehensive resource for researchers, providing access to over 15 million de-identified electronic medical records. This data distribution process adheres to strict ethical guidelines and is subject to thorough review by an institutional ethics board. All extracted data files were encoded to prevent personal identification of patients, and it was not possible to identify actual patient numbers. Since the data utilized in this study did not include patients’ personal information, there was no risk of physical or psychological damage to the patients who were the study subjects.

### 2.2. Ethical Approval and Consent to Participate

This study was approved by the Institutional Review Board of the Catholic University of Korea (XC20WIDI0029, 27 March 2020). All research was performed in accordance with the Declaration of Helsinki. The requirement for informed consent was waived by the Institutional Review Board of the Catholic University of Korea (XC20WIDI0029, 27 March 2020).

### 2.3. Definition of Simple and Complex Systemic Diseases

Patients with only one disease were classified as having a single systemic disease, while those with two or more other diseases were classified as having complex systemic diseases. These classifications were generated for each of the five mentioned systemic disease groups.

### 2.4. Analysis of the Relationship between Systemic Diseases and Dental Diseases

**Figure 1:**
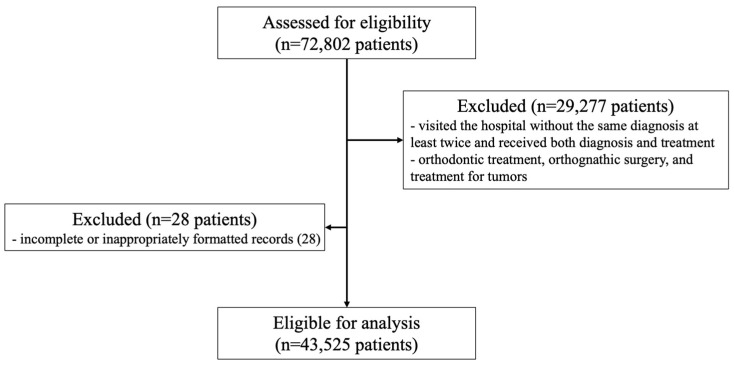
Flow diagram for the determination of the study population for assessment.

Of the 72,802 patients treated in the last 10 years, 43,563 were selected after the exclusion of patients who had received orthodontic treatment, orthognathic surgery, and treatment for tumors. A total of 28 patients were also excluded because they had incomplete or inappropriately formatted records; thus, data were collected from a final total of 43,525 patients (Figure 1). Patients with chronic diseases were selected as described above. The associations between each systemic disease and dental diseases were analyzed by comparing patients with and without systemic diseases. Additionally, the prevalence of dental diseases was compared between patients with multiple systemic diseases and those with only a single systemic disease.

### 2.5. Statistics

Statistical analyses were conducted using Python 3.8.8 to account for the complex sampling design and to provide nationally representative prevalence estimates. All data are presented as the mean ± standard error or proportion (standard error) for continuous or categorical variables, respectively. Statistical inference was implemented via the two-sample *t*-test and the two-sample Z-test for proportions embedded in the ‘statsmodels’ package, which evaluated whether two morbidity rates between patients with and without chronic diseases are different at several significance levels (α = 0.05, 0.01, 0.001, and 0.0001). We used multiple logistic regression analyses to examine the relationship between chronic/complex diseases and dental diseases. The adjusted odds ratios (ORs) and 95% confidence intervals for dental diseases were calculated for chronic diseases relative to the corresponding reference group. Calculations were made adjusting for age and sex, and *p*-values of less than 0.05 were considered statistically significant.

## 3. Results

**Figure 2:**
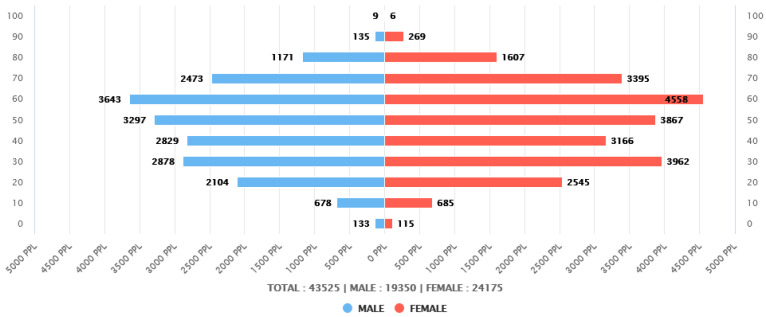
Distribution of included patients by sex and age.

Among the 72,802 people treated at the dental clinics of Seoul St. Mary’s Hospital and Eunpyeong St. Mary’s Hospital between 1 July 2011 and 30 June 2020, 43,525 patients were selected. Of these 43,525 patients, 19,350 were men (44.46%) and 24,175 were women (55.54%). Patients in their 60s represented the largest group, totaling 8,201 (18.84%), followed by those in their 50s with 7164 (16.46%). In contrast, patients aged 80 or older made up the smallest group, with 3198 (7.35%) (Figure 2 and Table 2). Patients were relatively evenly distributed by age, with no statistically significant differences among age groups.

The number of patients with at least one systemic disease was 14,111. The most common single systemic disease was HBP (n = 7478), followed by DM (n = 6828), OPR (n = 4688), RA (n = 3418), and DMT (n = 1015). The most common dental disease was periodontal disease (n = 10,497), followed by dental caries (n = 5241), pulp necrosis (n = 4735), temporomandibular joint disorder (TMD; n = 1531), tooth loss (n = 1530), and others (n = 680) (Table 3).

**Figure 3:**
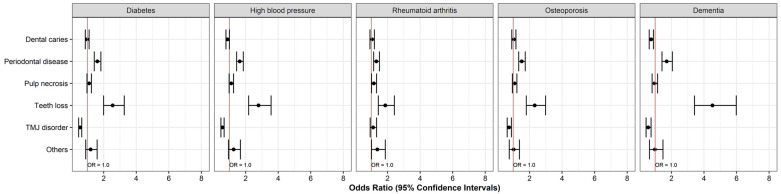
Odds ratios of single systemic diseases for dental disease. The red line represents Odds Ratio = 1.0. Values to the right indicate a higher observation of the dental diseases.

**Figure 4:**
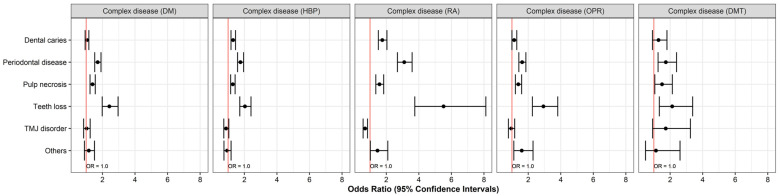
Odds ratios of complex systemic diseases for dental disease. The red line represents Odds Ratio = 1.0. Values to the right indicate a higher observation of the dental diseases.

Differences in morbidity according to sex were also observed. Male patients had higher rates of periodontal disease and pulp necrosis than female patients, and TMD was more common in women than in men. The systemic disease group showed higher odds of periodontal disease (OR = 1.708) and tooth loss (OR = 4.520) than the group without systemic disease but a lower likelihood of TMD morbidity (OR = 0.575). The morbidity of dental diseases was higher in patients with complex systemic diseases than in patients with DM, HBP, and DMT in isolation. The morbidity of dental diseases, excluding TMD, was higher in patients with complex systemic diseases than in patients with RA and OPR as single systemic diseases. The morbidity of tooth loss was especially high in the single DMT group (OR = 4.520) and in the complex RA group (OR = 5.519). Elsewhere, the risk of TMD was relatively low, except for in the complex DMT group (OR = 1.731) (Figure 3 and Figure 4).

**Figure 5:**
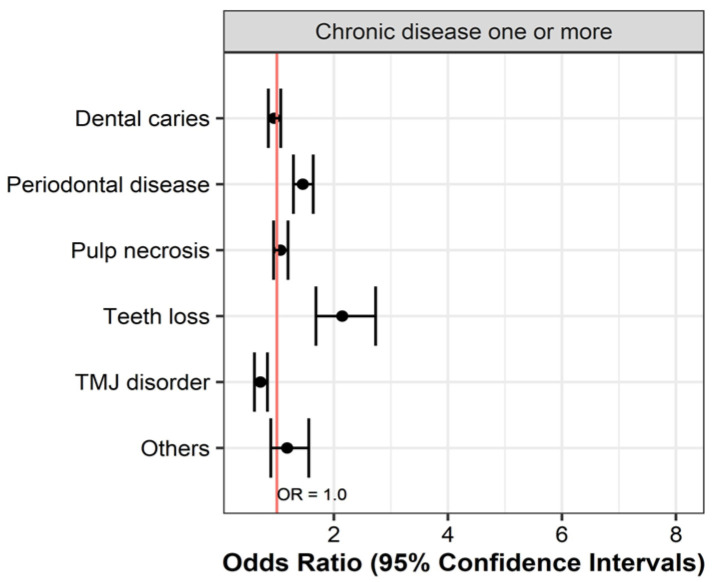
Odds ratios of single and complex systemic diseases for dental diseases. The red line represents Odds Ratio = 1.0. Values to the right indicate a higher observation of the dental diseases.

When all systemic disease groups were integrated and analyzed, the highest odds ratio was found for tooth loss (OR = 2.146), followed by periodontal disease (OR = 1.454). However, a lower risk of TMD was found (OR = 0.711). No statistically significant differences in risk were found for pulp necrosis, dental caries, and other dental diseases (Figure 5).

## 4. Discussion

This study investigated the relationships between systemic diseases—alone or in combination—and dental diseases using the CDW of the Catholic Medical Center of Korea. In this study, we found that subjects with one or more systemic diseases (DM, HBP, RA, OPR, and DMT) were at a higher risk for periodontal disease, pulp necrosis, and tooth loss. All systemic disease groups showed an elevated risk of periodontal disease, pulp necrosis, and tooth loss, but the RA and OPR group showed lower levels of TMD risk.

Periodontal disease, pulp necrosis, and tooth loss all showed significantly higher prevalence rates in patients with chronic diseases and complex chronic diseases. Regarding the correlation between periodontal disease, tooth loss, and chronic diseases, several previous studies reported similar results. Diabetes and OPR have been suggested as related chronic systemic diseases [14]. It was also reported that diabetes and cardiovascular disease are closely related to periodontal disease [15]. Periodontal infection with *Porphyromonas gingivalis* constitutes an inherent risk factor for the development of autoimmune antibodies associated with RA. Of note, many patients with RA lose multiple teeth or have advanced periodontitis [16]. A recent study examined the relationship between periodontal disease treatment and healthcare costs for patients with DM in commercial insurance and Medicaid claims data and found that undergoing periodontal treatment was associated with reduced overall and outpatient healthcare costs in both insurance types [17].

Many reports have also stated that HBP is closely related to dental diseases, such as periodontal disease and tooth loss. A previous study showed that decreased masticatory function, poor oral hygiene, and oral inflammation were associated with HBP [18]. Tooth loss was found to be associated with high systolic blood pressure in adults [19].

Patients with other chronic diseases showed significantly higher likelihoods of having periodontal disease, pulp necrosis, and tooth loss but a lower risk of having TMD, except for the RA group, which presented a higher rate of TMD. This is presumed to be because RA, as a joint disease with autoimmune characteristics, also affects the temporomandibular joint (TMJ) [20]. Therefore, the following three hypotheses can be proposed. First, in RA patients, it was found that the articular surface of the condyle is covered by inflammatory granulation, which may cause destruction of the osseous TMJ structures [21]. Second, RA patients’ symptoms are typically treated by immunosuppressive drugs and non-steroidal anti-inflammatory drugs. The use of these drugs and the increased bone resorption by RA could induce a significant deterioration of the anatomic TMJ structures [22]. Third, masticatory muscles might be affected by the bruxism and clenching caused by the psychological problems associated with RA [23]. Although only the RA group (OR = 1.110) and the complex DMT group (OR = 1.731) had an elevated risk of TMD in this study, this finding should be noted when considering treatment for patients with RA or DMT.

A noteworthy finding of this study is that the DMT group showed a significantly higher rate of tooth loss, and the combination of DMT with other chronic diseases was associated with a higher risk of all dental diseases. DMT is a geriatric disease, and it is reasonable to infer that these findings reflect the effects of aging. A recent meta-analysis showed a correlation between periodontitis and cognitive impairment, and moderate or advanced periodontitis was identified as a possible risk factor for DMT [24]. A recent systematic review found that elderly people with DMT had a higher incidence of dental caries and retained roots, which can cause orofacial pain [25]. Moreover, aged people with DMT usually have oral hygiene problems due to both age and DMT [26]. No consensus exists regarding whether this association reflects the effects of old age as a cause of cognitive and motor decline, or whether tooth loss affects DMT. Nonetheless, since severe dental diseases can coexist with DMT, special dental care is needed for these patients.

The OPR group showed a higher prevalence of tooth loss. OPR reduces the density of jaw bones and leads to fewer teeth, but it does not affect other clinical and laboratory signs of periodontitis such as inflammation, bleeding, the periodontal pocket depth, and plaque accumulation [27]. A prior study found that older women with OPR were more likely to also have periodontitis, with more prominent severity than those with normal bone mineral density, and proper OPR medication can prevent periodontitis [28]. Another study discussed the importance of oral health maintenance in patients with OPR. OPR and related fractures are common and can severely impact quality of life, cause functional impairment, and increase healthcare costs and mortality. Medical management of OPR includes medication, such as bisphosphonates, which have been associated with the development of osteonecrosis of the jaw. Therefore, dental professionals should be aware of the implications of bisphosphonate therapy and work with the patient’s physician to ensure appropriate management [29].

The relationship between various dental and systemic diseases has been explained in many ways, but, more recently, there is a growing view that the oral microbiome plays a key role in these connections. For example, interactions between the oral microbiome and systemic diseases such as diabetes and cardiovascular conditions are well established, with oral dysbiosis contributing to systemic inflammation [30]. Similarly, it has been shown that oral pathogens can exacerbate diseases like neurodegenerative disorders through inflammatory pathways [30]. Another review highlights the clear correlation between oral microbiota imbalance and diseases like diabetes and chronic inflammatory diseases, suggesting a significant role of dysbiosis in worsening these conditions [31]. As research continues to uncover the causal links between these diseases, there is an increasing need to focus on dental disease treatment to mitigate broader health impacts.

In this study, we identified significant associations between systemic diseases and dental health, with a particular emphasis on tooth loss. The findings reveal that DMT patients face a dramatically higher risk of tooth loss (OR = 4.520), even when dealing with just one systemic condition. Moreover, the risk becomes more pronounced in patients with multiple conditions, such as those with RA, where the odds of tooth loss increase even further (OR = 5.519). Among patients with any chronic disease, tooth loss emerged as the leading concern (OR = 2.146), followed by periodontal disease (OR = 1.454), suggesting that the presence of chronic systemic diseases significantly elevates dental risks. These insights underline the need for specialized care to prevent tooth loss in patients with dementia and RA, especially as chronic diseases exacerbate dental health challenges. This finding emphasizes the need for regular dental check-ups and periodontal treatment for those affected, along with education for healthcare providers to improve patient outcomes. This cross-sectional study has limitations inherent in its design, particularly its focus on five systemic diseases and a predominantly elderly Korean cohort, where 40% of participants were elderly and disproportionately affected by comorbid conditions. As a result, the findings may be subject to regional- and age-related biases. Future research should utilize longitudinal designs and include a more diverse age range and geographic representation to better capture the temporal relationships between systemic and dental diseases across varied populations.

## 5. Conclusions

This study provides valuable insights into the relationship between systemic and dental diseases in an elderly Korean population. Our findings show that patients with systemic diseases, particularly those with multiple conditions, are at significantly higher risk for developing dental diseases such as periodontal disease, pulp necrosis, and tooth loss. These associations were particularly strong in patients with diabetes, rheumatoid arthritis, and dementia, highlighting the need for integrated healthcare approaches.

A key contribution of this study is the identification of a higher prevalence of tooth loss in patients with dementia, underscoring the importance of specialized dental care in this group. Additionally, patients with multiple systemic diseases, such as rheumatoid arthritis, face a greater risk of severe dental issues compared to those with a single disease, emphasizing the importance of targeted interventions. Furthermore, patients with multiple systemic diseases face a greater risk of severe dental issues compared to those with a single systemic disease, emphasizing the need for targeted interventions.

While these associations have been suggested in previous research, our study expands on the existing literature by focusing on a large, elderly cohort and examining the compounding effects of multiple systemic diseases on dental health. However, as this study is cross-sectional, future longitudinal research is necessary to explore these relationships further and evaluate the impact of integrated care strategies.

## Figures and Tables

**Table 1:** Systemic diseases and dental diseases analyzed in this study.

Systemic Diseases	Dental Diseases
Diabetes	Dental caries
Hypertension	Periodontal disease
Rheumatoid arthritis	Pulp necrosis
Osteoporosis	Tooth loss
Dementia	Temporomandibular joint disorder
	Others (dental diseases other than those mentioned above)

**Table 2:** Distribution of included patients by sex and age.

		N (Total 43,525)	%
sex	Male	19,350	44.46
	Female	24,175	55.54
age	20–29	4649	10.68
	30–39	6840	15.72
	40–49	5995	13.77
	50–59	7164	16.46
	60–69	8201	18.84
	70–79	5868	13.48
	≥80	3198	7.35

**Table 3:** Distribution of dental disease according to chronic disease status.

		Total N	Dental Caries	Periodontal Disease	Pulp Necrosis	Tooth Loss	TMJ Disorder	Others
Chronic disease one or more	Yes	14,111	5241 (37.1)	10,497 (74.4)	4735 (33.6)	1530 (10.8)	1531 (10.8)	680 (4.8)
	No	1361	521 (38.3)	907 (66.6)	439 (32.3)	73 (5.4)	199 (14.6)	56 (4.1)
	OR (95% CIs)		0.953 (0.850–1.068)	1.454 (1.290–1.637)	1.061 (0.942–1.195)	2.146 (1.685–2.732)	0.711 (0.606–0.834)	1.180 (0.893–1.559)
	*p* value		0.406	<0.001	0.332	<0.001	<0.001	0.244
Diabetes (DM)	Yes	6828	2577 (37.7)	5209 (76.3)	2348 (34.4)	862 (12.6)	585 (8.6)	333 (4.9)
	No	1361	521 (38.3)	907 (66.6)	439 (32.3)	73 (5.4)	199 (14.6)	56 (4.1)
	OR (95% CIs)		0.977 (0.867–1.102)	1.610 (1.420–1.826)	1.101 (0.972–1.246)	2.549 (1.992–3.261)	0.547 (0.460–0.651)	1.195 (0.895–1.595)
	*p* value		0.708	<0.001	0.130	<0.001	<0.001	0.227
High blood pressure (HBP)	Yes	7478	2660 (35.6)	5727 (76.6)	2591 (34.6)	1021 (13.7)	666 (8.9)	384 (5.1)
	No	1361	521 (38.3)	907 (66.6)	439 (32.3)	73 (5.4)	199 (14.6)	56 (4.1)
	OR (95% CIs)		0.890 (0.791–1.003)	1.637 (1.445–1.855)	1.114 (0.984–1.260)	2.790 (2.184–3.564)	0.571 (0.481–0.677)	1.261 (0.947–1.680)
	*p* value		0.055	<0.001	0.087	<0.001	<0.001	0.111
Rheumatoid arthritis (RA)	Yes	3418	1345 (39.4)	2473 (72.4)	1214 (35.5)	326 (9.5)	546 (16)	190 (5.6)
	No	1361	521 (38.3)	907 (66.6)	439 (32.3)	73 (5.4)	199 (14.6)	56 (4.1)
	OR (95% CIs)		1.046 (0.919–1.190)	1.310 (1.145–1.499)	1.157 (1.012–1.322)	1.860 (1.432–2.418)	1.110 (0.931–1.323)	1.372 (1.011–1.861)
	*p* value		0.494	<0.001	0.032	<0.001	0.245	0.041
Osteoporosis (OPR)	Yes	4688	1828 (39)	3529 (75.3)	1590 (33.9)	545 (11.6)	532 (11.3)	197 (4.2)
	No	1361	521 (38.3)	907 (66.6)	439 (32.3)	73 (5.4)	199 (14.6)	56 (4.1)
	OR (95% CIs)		1.031 (0.910–1.166)	1.524 (1.338–1.737)	1.078 (0.947–1.226)	2.321 (1.804–2.986)	0.747 (0.628–0.890)	1.022 (0.755–1.384)
	*p* value		0.635	<0.001	0.253	<0.001	0.001	0.887
Dementia (DMT)	Yes	1015	323 (31.8)	785 (77.3)	320 (31.5)	207 (20.4)	91 (9)	41 (4)
	No	1361	521 (38.3)	907 (66.6)	439 (32.3)	73 (5.4)	199 (14.6)	56 (4.1)
	OR (95% CIs)		0.753 (0.634–0.893)	1.708 (1.419–2.056)	0.967 (0.812–1.151)	4.520 (3.414–5.983)	0.575 (0.442–0.748)	0.981 (0.650–1.480)
	*p* value		0.001	<0.001	0.706	<0.001	<0.001	0.927
Complex disease (DM)	Simple	1981	686 (34.6)	1266 (63.9)	547 (27.6)	123 (6.2)	154 (7.8)	78 (3.9)
	Complex	4847	1726 (35.6)	3642 (75.1)	1677 (34.6)	670 (13.8)	385 (7.9)	220 (4.5)
	OR (95% CIs)		1.044 (0.935–1.165)	1.707 (1.525–1.910)	1.387 (1.236–1.556)	2.423 (1.984–2.959)	1.024 (0.843–1.244)	1.160 (0.891–1.510)
	*p* value		0.442	<0.001	<0.001	<0.001	0.814	0.270
Complex disease (HBP)	Simple	2415	702 (29.1)	1534 (63.5)	698 (28.9)	188 (7.8)	213 (8.8)	114 (4.7)
	Complex	5063	1767 (34.9)	3816 (75.4)	1739 (34.3)	742 (14.7)	396 (7.8)	225 (4.4)
	OR (95% CIs)		1.308 (1.178–1.454)	1.758 (1.582–1.952)	1.287 (1.158–1.430)	2.034 (1.719–2.406)	0.877 (0.737–1.044)	0.939 (0.745–1.182)
	*p* value		<0.001	<0.001	<0.001	<0.001	0.140	0.591
Complex disease (RA)	Simple	1308	352 (26.9)	616 (47.1)	335 (25.6)	30 (2.3)	221 (16.9)	44 (3.4)
	Complex	2110	828 (39.2)	1549 (73.4)	743 (35.2)	242 (11.5)	262 (12.4)	102 (4.8)
	OR (95% CIs)		1.754 (1.510–2.038)	3.102 (2.683–3.586)	1.579 (1.355–1.840)	5.519 (3.751–8.117)	0.697 (0.574–0.847)	1.459 (1.017–2.092)
	*p* value		<0.001	<0.001	<0.001	<0.001	<0.001	0.039
Complex disease (OPR)	Simple	1430	497 (34.8)	900 (62.9)	388 (27.1)	69 (4.8)	156 (10.9)	39 (2.7)
	Complex	3258	1231 (37.8)	2392 (73.4)	1112 (34.1)	422 (13)	342 (10.5)	140 (4.3)
	OR (95% CIs)		1.140 (1.001–1.298)	1.627 (1.425–1.857)	1.392 (1.213–1.597)	2.935 (2.257–3.815)	0.958 (0.783–1.170)	1.602 (1.116–2.296)
	*p* value		0.048	<0.001	<0.001	<0.001	0.674	<0.001
Complex disease (DMT)	Simple	211	56 (26.5)	133 (63)	50 (23.7)	23 (10.9)	12 (5.7)	7 (3.3)
	Complex	804	255 (31.7)	601 (74.8)	256 (31.8)	166 (20.6)	76 (9.5)	30 (3.7)
	OR (95% CIs)		1.286 (0.915–1.806)	1.736 (1.259–2.394)	1.504 (1.060–2.136)	2.127 (1.335–3.387)	1.731 (0.923–3.248)	1.130 (0.489–2.609)
	*p* value		0.147	<0.001	0.022	0.001	0.084	0.775

## Data Availability

The data that support the findings of this study are available from the corresponding author upon reasonable request.
